# Supplementary figures and images for: Times and partners are a-changin’: relationships between declining food abundance, breeding success, and divorce in a monogamous seabird species
Source: PeerJ. 2022 Apr 8;10:e13073. doi: 10.7717/peerj.13073 (PMC8997194; doi:10.7717/peerj.13073)

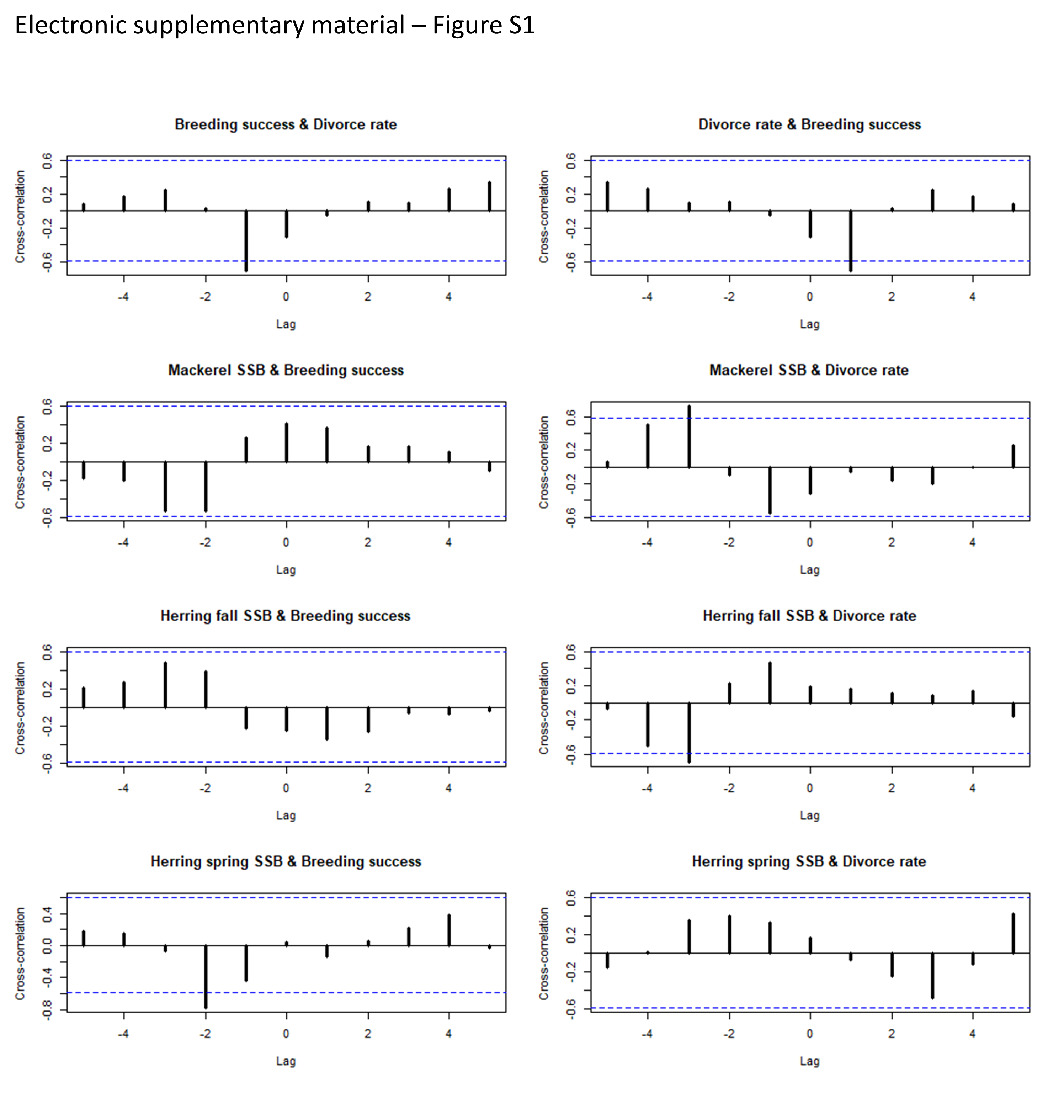

Supplement: Supplemental Information 1 [file peerj-10-13073-s001.png]
